# Effect of IL-1β on the Development of Spermatogenesis In Vitro in Normal and Busulfan-Treated Immature Mice

**DOI:** 10.3390/ijms25094926

**Published:** 2024-04-30

**Authors:** Nagham Ali, Eitan Lunenfeld, Mahmoud Huleihel

**Affiliations:** 1 The Shraga Segal Department of Microbiology, Immunology and Genetics, Faculty of Health Sciences, Ben-Gurion University of the Negev, Beer Sheva 8410501, Israel; nagham902@gmail.com; 2 The Center of Advanced Research and Education in Reproduction (CARER), Faculty of Health Sciences, Ben-Gurion University of the Negev, Beer Sheva 8410501, Israel; 3 Faculty of Health Sciences, Ben-Gurion University of the Negev, Beer Sheva 8410501, Israel; 4 Adelson School of Medicine, Ariel University, Ariel 4070000, Israel; eitanlun@ariel.ac.il

**Keywords:** male infertility, spermatogenesis, chemotherapy, in vitro differentiation of spermatogonial cells, Sertoli cells, Leydig cells

## Abstract

Gonadotoxic agents could impair spermatogenesis and may lead to male infertility. The present study aimed to evaluate the effect of IL-1β on the development of spermatogenesis from cells isolated from seminiferous tubules (STs) of normal and busulfan-treated immature mice in vitro. Cells were cultured in a 3D in vitro culture system for 5 weeks. We examined the development of cells from the different stages of spermatogenesis by immunofluorescence staining or qPCR analyses. Factors of Sertoli and Leydig cells were examined by qPCR analysis. We showed that busulfan (BU) treatment significantly reduced the expression of testicular IL-1β in the treated mice compared to the control group (CT). Cultures of cells from normal and busulfan-treated immature mice induced the development of pre-meiotic (Vasa), meiotic (Boule), and post-meiotic (acrosin) cells. However, the percentage of developed Boule and acrosin cells was significantly lower in cultures of busulfan-treated mice compared to normal mice. Adding IL-1β to both cultures significantly increased the percentages of Vasa, Boule, and acrosin cells compared to their controls. However, the percentage of Boule and acrosin cells was significantly lower from cultures of busulfan-treated mice that were treated with IL-1β compared to cultures treated with IL-1β from normal mice. Furthermore, addition of IL-1β to cultures from normal mice significantly increased only the expression of androgen receptor and transferrin but no other factors of Sertoli cells compared to their CT. However, the addition of IL-1β to cultures from busulfan-treated mice significantly increased only the expression of androgen-binding protein and the FSH receptor compared to their CT. Adding IL-1β to cultures of normal mice did not affect the expression of 3βHSD compared to the CT, but it significantly reduced its expression in cultures from busulfan-treated mice compared to the CT. Our findings demonstrate the development of different stages of spermatogenesis in vitro from busulfan-treated mice and that IL-1β could potentiate this development in vitro.

## 1. Introduction

Spermatogenesis is the process of sperm production. This process begins with mitotic divisions of testicular stem cells that are located close to the basal membrane of the seminiferous tubules in the testis. These cells continue with meiotic divisions and eventually become sperm cells. Due to the high division rate of these cells, they are affected by chemotherapy and radiotherapy treatments, which may lead to male infertility [1].

Busulfan (BU) is a cytotoxic drug, an alkylating agent, and is used in the treatment of cancer and certain cases of bone marrow transplantation [1]. The drug damages all types of proliferating cells, including those in the reproductive system. Busulfan affects the proliferating testicular cells [2,3]. In addition, it may affect the functionality of testicular somatic cells and the microenvironment (niches) of the cells in the seminiferous tubules [2,3,4]. The microenvironment includes the endocrine and paracrine/autocrine factors that control spermatogenesis; therefore, any changes in the microenvironment may impair the development of the spermatogenetic process [2,3,4].

Testicular autocrine/paracrine regulation controls the process of spermatogenesis locally by secreting various cytokines and growth factors involved in the process. They are involved in regulation of the proliferation and differentiation of germ cells, as well as the function of somatic cells (Sertoli and Leydig cells) [5,6]. Interleukin-1 (IL-1) is one of the autocrine/paracrine factors that exist in the testis under physiological conditions [7].

Interleukin-1 is a pro-inflammatory cytokine and an immunoregulatory polypeptide growth factor [7]. It is produced mainly by macrophages in response to foreign antigens, pathogens, and chronic inflammations. The IL-1 family can induce growth, inhibition, and differentiation of different cells in the body and are autocrine/paracrine factors with pleiotropic activity [7,8]. The most studied members of the IL-1 family are IL-1α, IL-1β, and the IL-1 receptor antagonist (IL-1Ra). IL-1β is present in various testicular cells, such as the interstitial cells (Leydig cells and testicular macrophages) and tubular cells (Sertoli cells and germ cells at different stages of differentiation), under physiological conditions [9,10,11]. IL-1β was shown to affect the proliferation of germ cells and Leydig cells [12,13]. The levels of IL-1β in the testis were shown to be affected (mainly increased) following pathological conditions, such as LPS treatment among others [13]. Previous studies showed that injecting busulfan into mouse testes increases the levels of cytokines, including IL-1β [14,15].

The functionality of somatic cells (Sertoli and Leydig) in the testis is regulated mainly by FSH and LH [16]. LH affects the production of testosterone by Leydig cells in the interstitial tissue of the testis, which controls the process of spermatogenesis [17,18]. FSH binds to the FSH receptor on Sertoli cells which controls their activity. It induces the secretion of essential factors for the development and maturation of spermatogonial cells in the seminiferous tubules, such as androgen-binding protein (ABP), the androgen receptor (AR), GDNF, and others [19,20]. Paracrine factors and cytokines could also affect the functionality of those somatic cells [21].

Infertility could be a serious side effect of chemotherapy. Adult males can cryopreserve their sperm before treatment for future reproduction using assisted reproductive technologies [22]. This option is not feasible for prepubertal boys who are not yet producing mature sperm. They do, however, have spermatogonial stem cells (SSCs) in the testis that are poised to initiate spermatogenesis at puberty. In vitro, maturation of spermatogonial cells is one of the techniques that are currently in the research pipeline and part of the effort to preserve and restore fertility for those that undergo chemo/radiotherapy at the prepubertal age [22]. Various factors are involved in the regulation of the development of the spermatogenesis process under in vivo and in vitro conditions; however, the specific testicular autocrine/paracrine factors involved in each process (growth, proliferation, and differentiation) are still not completely clear. Finding out these factors may provide optimal conditions to grow spermatogonial cells in culture. Currently, different methods and distinct growth conditions with a wide variety of growth media and sera are being evaluated [23,24,25]. Even though some success has been achieved in terms of inducing the development of some stages of spermatogenesis in vitro [26], protocols for constant induction of complete spermatogenesis in vitro, starting from spermatogonial cells, are not yet available.

In this study, we used immature mice to mimic the effect of gonadotoxic agents during prepuberty on the development of spermatogenesis in the adult age and their effect on factors that are involved in normal spermatogenesis. We also used immature mice (before the development of spermatogenesis) to examine the development of different stages of spermatogenesis in vitro. We examined the effect of busulfan treatment among immature male mice on the expression levels of IL-1β in their testes during development into adulthood. Furthermore, we examined the effect of IL-1β on the development of spermatogenesis from spermatogonial cells of normal immature mice and busulfan-treated immature mice using a three-dimension methylcellulose in vitro culture system.

## 2. Results

### 2.1. Localization and Expression Levels of IL-1β in the Testes of Normal Mice Compared to Busulfan-Treated Mice

**Figure 1:**
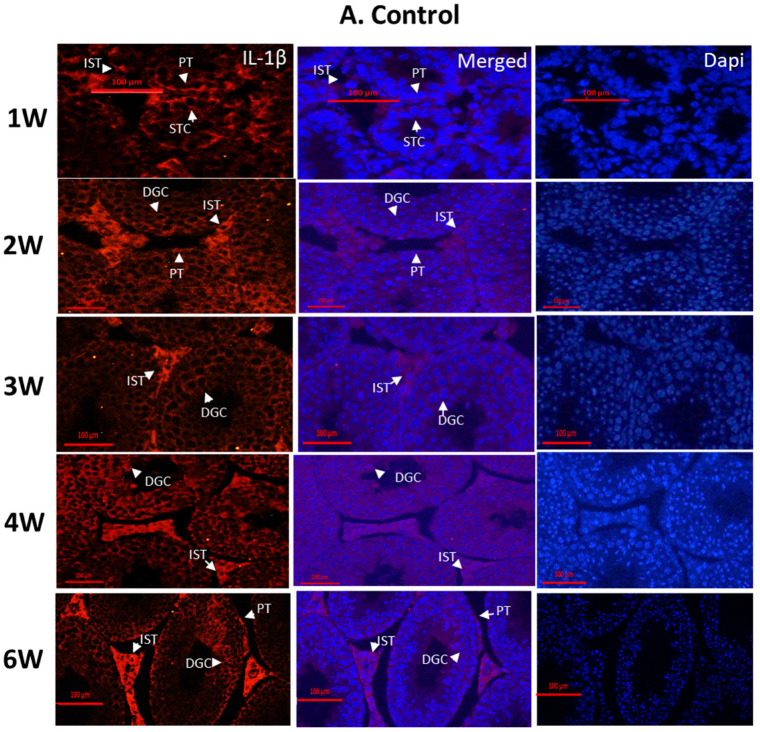

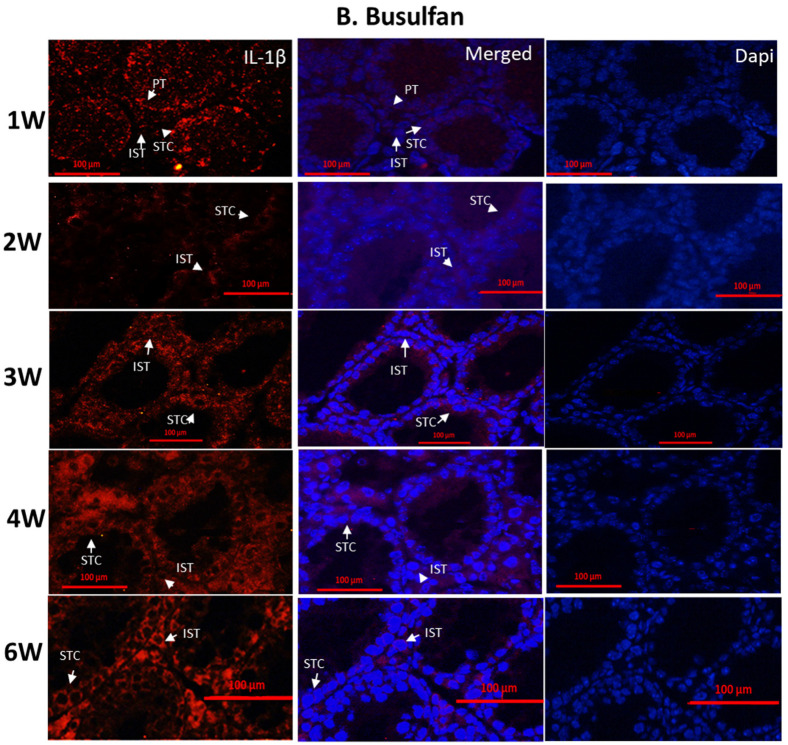
Effect of busulfan on the localization of IL-1β in mouse testicular cells. Seven-day-old mice were intraperitoneally injected (IP) with busulfan (BU) or DMSO (control group (CT)) as described in the Section 4. Testes were collected 1, 2, 3, 4, and 6 weeks post injection. Testes from control mice (control) (**A**) or from busulfan-injected mice (busulfan) (**B**) were fixed in Bouin’s solution, sectioned, and stained by immunofluorescence (IF) staining for IL-1β using specific antibodies. Arrows indicate staining of IL-1β in interstitial cells (ISTs), germ cells (GCs), germ cells at different stages of development (differentiated germ cells; DGCs), or peritubular cells (PTs). The testicular tissues were counterstained with DAPI to indicate nucleus localization. The tissues shown present DAPI, IL-1β, and merge staining.

To examine the cellular location and the expression levels of IL-1β in testicular cells from normal/busulfan-treated mice, we collected testes from normal and busulfan-treated mice at different time points post injection (1–6 weeks post injection). To characterize the cell types that express IL-1β in both physiological and pathological conditions, the collected testis tissues were fixed and used for immunofluorescent staining of IL-1β. IL-1β was present in most testicular cell types in the seminiferous tubules (peritubular cells—PTs; germ cells—GCs) and the interstitial cells (ISTs) in both control and busulfan-treated mice (Figure 1A and Figure 1B, respectively). In the control group, IL-1β was identified in the advanced stages of differentiation of germ cells (developing germ cells—DGCs) (Figure 1A); those developing germ cells (DGCs) were depleted following busulfan treatment (Figure 1B). In addition, our results show that IL-1β is mainly present in the interstitial compartment (IST) composed of Leydig cells and macrophages in both the control and busulfan-treated groups (Figure 1A and Figure 1B, respectively).

**Figure 2:**
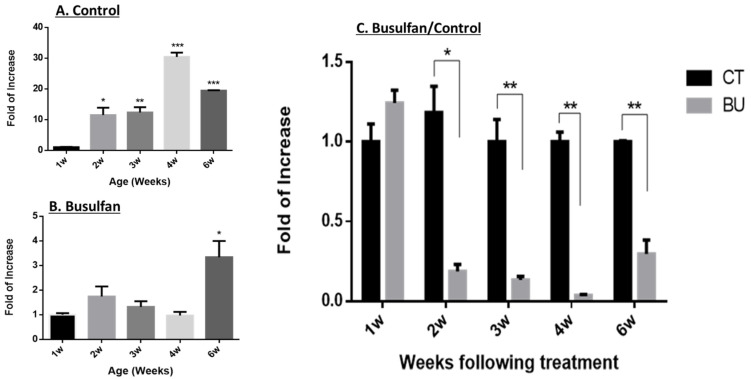
Effect of busulfan on the expression levels of IL-1β in mouse testicular homogenates. Mice were injected with busulfan or DMSO as described in Figure 1. The expression levels of IL-1β in testicular homogenates of the control group (CT) (**A**) or busulfan injected group (busulfan) (**B**) at the different time points (1–4 and 6 weeks post injection) were examined by qPCR analysis using specific primers for IL-1β. We also compared the expression levels of IL-1β in testicular homogenates of busulfan-injected mice to control mice (**C**). GAPDH was used as a housekeeping gene (N (BU) = 4, N (CT) = 4 (N = number of repeats), n = 10 (n = number of mice/N), *p*-value (* *p* < 0.05; ** *p* < 0.01; *** *p* < 0.0001)).

In parallel to immunohistochemistry, we also examined IL-1β mRNA expression levels in those testes using qPCR analysis (Figure 2). Our results show that the expression levels of IL-1β in the control group significantly increased with age at all examined time points (2–6 weeks compared to the first week). The highest expression level of IL-1β was demonstrated after 4 weeks (Figure 2A). However, in busulfan-treated mice, IL-1β expression levels increased significantly only 6 weeks after injection compared to the first week (Figure 2B). Moreover, the expression levels of IL-1β in the testes of busulfan-treated mice significantly decreased compared to normal mice at all time points post injection, excluding the first week post injection, which showed similar expression levels between the control and the busulfan group (Figure 2C).

### 2.2. Involvement of IL-1β in the In Vitro Development of Spermatogenesis from Cells of Seminiferous Tubules in Normal and Busulfan-Treated Immature Mice

#### 2.2.1. Identification of Developed Cells in the Methylcellulose Culture System (MCS)

**Figure 3:**
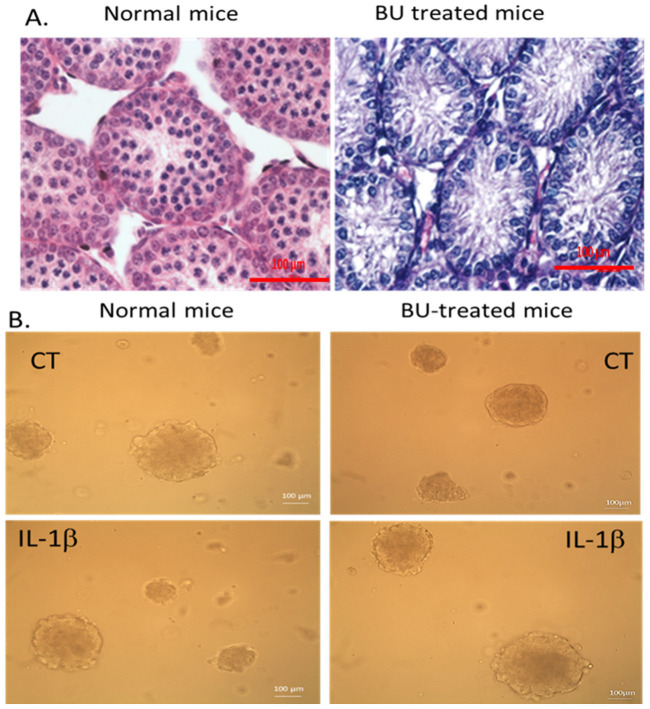

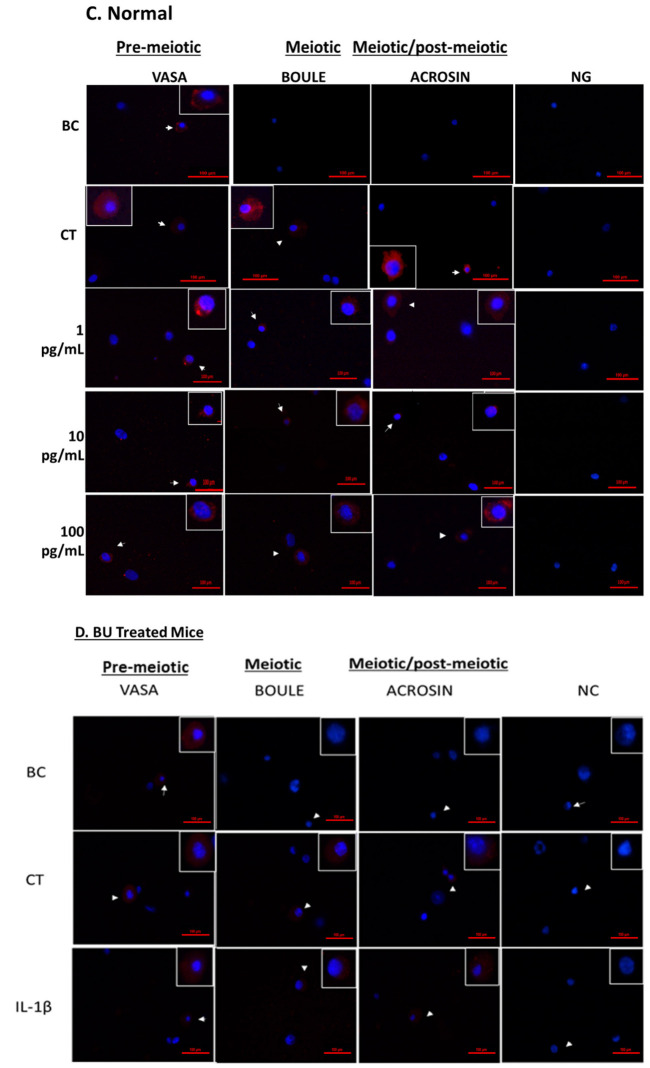
Histology of testes from normal and busulfan-treated mice and identification of developed cells in the MCS. Testes from immature mice (7-day-old) (normal mice; **A**) or from busulfan-injected mice (10 days post injection of the immature mice) (BU-treated mice; **B**) were fixed in Bouin’s solution and stained with hematoxylin–eosin for histological examination. Cells were enzymatically isolated from the testes of mice described above and were cultured for 5 weeks in methylcellulose 3D culture (MCS) containing StemPro Medium, various growth factors, and KSR 10% (see Section 4 in the absence (CT) or presence of IL-1β (1, 10, and 100 pg/mL) (IL-1β). The morphologies of cultures (from normal and BU-treated mice) in the absence (CT) or presence of IL-1β (IL-1β) after 5 weeks show the presence of cells and organoids (**B**). Developed cells from normal (**C**) and busulfan-treated mice (**D**) were collected after 5 weeks, fixed, and stained with specific antibodies to markers specific for the different stages of spermatogenesis, such as the pre-meiotic marker (Vasa and GFRα), meiotic marker (Boule), and post-meiotic marker (acrosin). DAPI (blue) was used for nucleus staining (NC, negative control; BC, before culture; CT, control (without IL-1β)). Cultures were grown in the presence of different concentrations of IL-1β (1, 10, 100 pg/mL) (IL-1β). The number of repeated experiments was (N) = 4–5, and the number of wells for each treatment in each experiment was (*n*) = 3. We present pictures representative of the experiments.

Following our finding of the localization of IL-1β in different testicular cells and the reduction in its expression following busulfan treatment, we evaluated its possible involvement in the development of spermatogenesis in vitro. We isolated cells from seminiferous tubules of normal immature mice (7-day-old) or busulfan-treated immature mice (8–10 days post injection) and cultured them in vitro in an MCS in the presence or absence of IL-1β. Our results show that injection of busulfan caused damage to the histological structure of the seminiferous tubules compared to control mice (Figure 3A). In addition, our results show that isolated cells from seminiferous tubules of normal and busulfan-treated immature mice developed organoids in the MCS after 5 weeks of culture in the absence (CT) or presence of IL-1β (IL-1β) (Figure 3B). In addition, our results show that the organoids developed in both types of cultures (control and busulfan-treated mice), and the absence or the presence of IL-1β did not lead to a significant difference in the size, structure, or quantity of those organoids (Figure 3A). Developed cells and organoids in the MCS in the absence or presence of different concentrations of IL-1β (1, 10, 100 pg/mL) were collected after 5 weeks of culture to identify the type of spermatogenic cells that developed in the cultures (identified by specific immunofluorescence staining) and the expression levels of the markers of these cells (identified by qPCR analysis). To identify the cell types expressed in the cultures of control (Figure 3C) and busulfan-treated immature mice (Figure 3D), the collected cells were fixed and stained with specific antibodies for cell markers of the premeiotic (Vasa), meiotic (Boule), and post-meiotic (acrosin) stages of spermatogenesis. Developed organoids, cells, and cells before culture (BC) from control (Figure 3C; BC) and from busulfan-treated mice (Figure 3D; BC) stained positively only for Vasa but not for Boule or acrosin as expected. However, cells from cultures of control immature mice in the absence (CT) or presence of different concentrations of IL-1β (Figure 3C) and from busulfan-treated mice in the absence (CT) or presence of IL-1β (Figure 3D) were positively stained for Vasa, Boule, and acrosin.

#### 2.2.2. Cultures from Normal Mice—Quantification of the Effect of IL-1β on the Percentages of Developed Spermatogenic Cells and the Expression Levels of Their Markers

**Figure 4:**
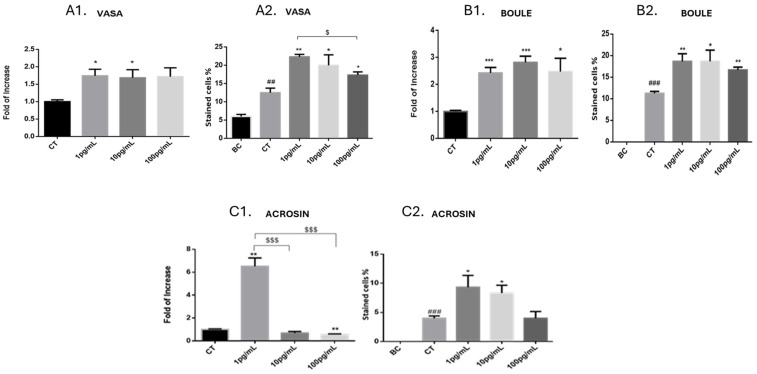
Quantification of the effect of IL-1β on the percentages of developed spermatogenic cells and the expression levels of their markers in cultures from normal mice. Testicular cells were isolated from seminiferous tubules of normal immature mice and cultured in an MCS (see Section 4) in the absence or presence of IL-1β (1, 10, and 100 pg/mL). Cultured cells were collected after 5 weeks and stained with Vasa (**A2**), Boule (**B2**), and acrosin (**C2**). In addition, RNA expression levels of Vasa (**A1**), Boule (**B1**), and acrosin (**C1**) were examined in these cultures by qPCR analysis using specific primers for each factor. GAPDH was used as a housekeeping gene. CT, control (without IL-1β); BC, before culture. The number of repeated experiments was (N) = 10, and the number of wells for each treatment in each experiment was (n) = 8. * compared to CT. # compared to BC. *p*-values: * *p* < 0.05, ** *p* < 0.01, *** *p* < 0.0001, ## *p* < 0.01, ### *p* < 0.001. $ *p* <0.05, $$$ *p* < 0.001.

Our results showed that the expression levels (Figure 4A1) and the percentages (Figure 4A2) of the pre-meiotic cell marker Vasa and the meiotic marker Boule (Figure 4B1 and Figure 4B2, respectively) were significantly increased in cultures of cells from normal immature mice in the presence of IL-1β (in a dose-dependent manner) compared to the control group (CT (without IL-1β)). Furthermore, the percentages of Vasa- and Boule-stained cells were significantly higher in control cultures (CT) compared to before culture (BC) (Figure 4A2 and Figure 4B2, respectively). On the other hand, the expression levels of acrosin were significantly increased only in the lower concentration of IL-1β in the culture (1 pg/mL) and decreased with increasing IL-1β concentration (100 pg/mL) compared to the control (CT (without IL-1β)) (Figure 4C1). However, the percentages of acrosin-positive cells significantly increased in culture compared to before culture (BC), as well as in the presence of IL-1β (1 and 10 pg/mL) compared to the control (CT) (Figure 4C2).

#### 2.2.3. Cultures from Busulfan-Treated Immature Mice—Quantification of the Effect of IL-1β on the Percentages of Developed Spermatogenic Cells and the Expression Levels of Their Markers

**Figure 5:**
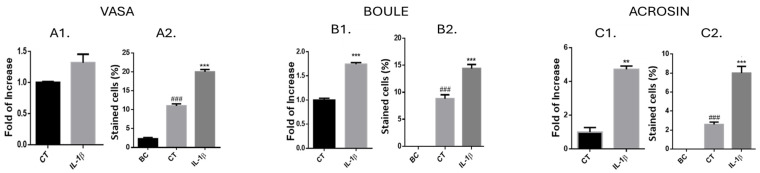
Quantification of the effect of IL-1β on the percentages of developed spermatogenic cells and the expression levels of their markers in cultures from busulfan-treated mice. Testicular cells were enzymatically isolated from seminiferous tubules of busulfan-treated immature mice and cultured in an MCS as described in Figure 4. Cultured cells were collected after 5 weeks and stained with Vasa (**A2**), Boule (**B2**), and acrosin (**C2**). In addition, RNA expression levels of Vasa (**A1**), Boule (**B1**), and acrosin (**C1**) were examined in these cultures through qPCR analysis using specific primers for each factor. GAPDH was used as a housekeeping gene. CT, control (without IL-1β); BC, before culture. The number of repeated experiments was (N) = 4, and the number of wells for each treatment in each experiment was (n) = 8. * compared to CT. # compared to BC. *p*-values: ** *p* < 0.01, *** *p* < 0.0001, ### *p* < 0.001.

As shown in our above results (Figure 4) using cell cultures from normal immature mice, we stimulated cells that were cultured from busulfan-treated immature mice only in the presence of one concentration of IL-1β (1 pg/mL), which led to an optimal effect on the development of spermatogenic cells in the control group without busulfan (Figure 4). Our results show that the expression levels and the percentages of the pre-meiotic cell marker Vasa (Figure 5A1 and Figure 5A2, respectively), the meiotic marker Boule (Figure 5B1 and Figure 5B2, respectively), and the post-meiotic marker (acrosin) significantly increased in cultures of cells from busulfan-treated immature mice in the presence of IL-1β compared to the control group (CT (without IL-1β)). Furthermore, the percentages of Vasa-, Boule-, and acrosin-stained cells were significantly higher in control (CT) cultures compared to before culture (BC) (Figure 5A2, Figure 5B2, and Figure 5C2, respectively).

### 2.3. Summary of the Effect of IL-1β on the Percentages of Spermatogenic Cells Developed In Vitro from Cultures of Cells Isolated from Seminiferous Tubules of Normal and Busulfan-Treated Immature Mice

Our results show that the prevalence of Vasa-positive stained cells was significantly lower in cells isolated from seminiferous tubules of busulfan-treated mice compared to normal mice (Table 1). However, the percentages of Vasa-positive stained cells were similar after 5 weeks of culture for normal and busulfan-treated mice (BU) in the CT (in the absence of IL-1β) and in the presence of IL-1β (Table 1). Furthermore, the prevalence of Vasa-positive stained cells was significantly higher in cultures with IL-1β compared to control (CT) cultures for both normal and busulfan-treated immature mice (Table 1).

Our results also show the absence of Boule- or acrosin-positive stained cells in cells isolated from seminiferous tubules of normal and busulfan-treated immature mice before culture (BC) (Table 1). However, the percentages of Boule- and acrosin-positive stained cells were significantly lower after 5 weeks of cultures in busulfan-treated immature mice (BU) compared to normal immature mice in the CT (in the absence of IL-1β) and in the presence of IL-1β (Table 1). In addition, for busulfan-treated immature mice, the percentages of Boule- or acrosin-positive stained cells were significantly higher after 5 weeks of culture when treated with IL-1β compared to control (CT) cultures (Table 1).

### 2.4. Effect of IL-1β on the Functionality of Sertoli and Leydig Cells In Vitro from Normal and Busulfan-Treated Immature Mice

**Figure 6:**
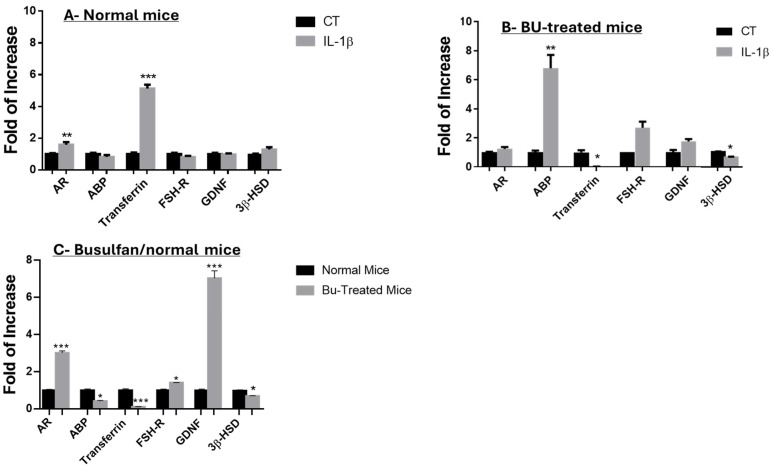
Effect of IL-1β on the functionality of Sertoli and Leydig cells in vitro of normal and busulfan-treated mice. Testicular cells were isolated from seminiferous tubules of normal and busulfan-treated immature mice and cultured in 3D cultures as mentioned in Figure 4 and Figure 5. Cultured cells from normal mice or busulfan-treated mice (**A**–**C**) were collected after 5 weeks and examined for the expression levels of factors produced by Sertoli cells, such as the androgen receptor (AR), androgen-binding protein (ABP), glial cell-derived nerve growth factor (GDNF), transferrin, and the FSH receptor (FSH-R), or for the functional marker of Leydig cells (3beta hydroxy steroid dehydrogenase; 3βHSD). The differences in expression between busulfan-treated mice and normal mice are presented in (**C**). RNA expression levels were examined through qPCR analysis using specific primers for each factor. GAPDH was used as a housekeeping gene. CT signifies the control group (without IL-1β). The number of repeated experiments was (N) = 10, and the number of wells for each treatment in each experiment was (n) = 4. The significance values reflect the relation between IL-1β treatment and the CT for each marker. *p*-values: * *p* < 0.05, ** *p* < 0.01, *** *p* < 0.0001.

To deepen our understanding of the effect of IL-1β in the culture, we examined the expression levels of factors that specifically indicate the functionality of Sertoli cells, such as AR, ABP, transferrin, FSH-R, and GDNF (Figure 6A,B). Our results show that the addition of IL-1β to isolated cells from seminiferous tubules of normal mice significantly increased the expression levels of AR and transferrin without significant effects on ABP, FSH-R, or GDNF compared to the control group (CT) (Figure 6A). However, the addition of IL-1β to isolated cells from seminiferous tubules of busulfan-treated mice significantly increased the expression levels of ABP and FSH-R without a significant effect on AR or GDNF, though there was a significant decrease in transferrin levels compared to the control group (CT) (Figure 6B).

The expression levels of 3bHSD (Leydig cell marker) exhibited insignificant changes after the addition of IL-1β to the culture of normal mice (Figure 6A), while a significant decrease in 3βHSD was demonstrated after addition of IL-1β to the culture of busulfan-treated mice (Figure 6B). Furthermore, our results show that cultures from busulfan-treated immature mice significantly increased the expression levels of AR, FSH-R, and GDNF but significantly decreased the expression levels of ABP, transferrin, and 3βHSD compared to cultures from normal mice (Figure 6C).

## 3. Discussion

Busulfan is an aggressive chemotherapy which may lead to male infertility and affects the proliferation of testicular cells. It also may lead to an imbalance among the different growth factors, which constitute part of the microenvironment in the testis. As a result, it may impair the development of normal spermatogenesis. In this study, we investigated the effect of chemotherapy (busulfan; BU) on the localization and expression levels of IL-1β as a growth factor present normally in mouse testicular tissue and involved in spermatogenesis [9,14]. We evaluated the direct and indirect effect of IL-1β on the capacity of spermatogonial cells isolated from normal and busulfan-treated immature mice to develop to different stages of spermatogenesis in vitro.

Treating young male mice with busulfan led to severe histological damage in the seminiferous tubules, depletion of developing germ cells, and a long-term reduction in IL-1β expression. Addition of IL-1β to the culture induced spermatogenesis in both normal and pathological conditions, although mice treated with busulfan showed a lower response to IL-1β.

The reduction in IL-1β expression levels post busulfan treatment may suggest a possible role for IL-1β in the impairment of spermatogenesis post busulfan therapy. IL-1β is also considered a growth factor expressed in the testis physiologically [14]. This decrease in its expression levels may be a result of a reduction in the types and numbers of cells in the seminiferous tubules and/or a suppression effect on the expression levels of IL-1β in its producing cells in the testis. It has been shown that IL-1β levels are affected in the testis following pathological conditions such as testicular torsion [13]. IL-1β may also be regulated by germ cells, and depletion of these cells following busulfan treatment may cause a decrease in its expression. It has been shown that germ cells play a major and widespread role in the regulation of somatic cell secretions and activities [22]. Moreover, it has been shown that busulfan has a direct effect on Sertoli cells by disturbing their differentiation [27]. Impairment of spermatogenesis could also be the cause for changes in the expression levels of IL-1β.

The different behavior (expression) of IL-1β was observed after the first week of chemotherapy compared to the other time points, which can be explained by the fact that there are differences in the types of germ cells in sexually immature mice that change over time after busulfan treatment, consequently causing a different regulation of the activity of Sertoli cells [4]. Our results show that the levels of IL-1β were significantly increased after 6 weeks of busulfan treatment; however, these levels were still significantly lower compared to the control group. This could be related to the absence of different types of germ cells (according to our histology) and to the impairment of Sertoli cells, Leydig cells, and macrophages, which are the main producers of IL-1 in the testis [8,9,11,17]. Our results are contrary to other studies that show a significant increase in testicular IL-1 following busulfan treatments (see the recent review by Lingjun Zhao et al.; 2023) [14,15]. This review and other studies show that the increase in the expression of IL-1 and other inflammatory cytokines is related to the development of inflammation in the testis. However, in our study we did not see (histologically) the development of inflammation in the testis. Thus, the absence of germ cells at different stages of development and possible impairment of the functionality of testicular somatic cells alongside the absence of inflammatory cells in the testis of busulfan-treated mice may explain the contradiction between our results and others.

The addition of IL-1β to cultures from both normal and busulfan-treated immature mice caused an increase in pre-meiotic (Vasa), meiotic (Boule), and post-meiotic (acrosin) cells. These findings indicate the involvement of IL-1β in regulation of the development process of spermatogenesis. An increase in pre-meiotic cells (Vasa) indicates the involvement of IL-1β in the proliferation stage, and an increase in meiotic (Boule) and post-meiotic cells (acrosin) indicates its possible involvement in the regulation of germ cell differentiation. Furthermore, our results show that adding low doses of IL-1β induces acrosin expression and stained cells, while high doses significantly decreased acrosin expression compared to the CT. This could be a result of negative regulation of IL-1β at high doses, thus affecting the development of acrosin in vitro. This effect could be direct on the process of developed acrosin cells and/or expressed through its effect on Sertoli and Leydig cells present in the culture. In addition, the addition of IL-1β in vitro distinctly affected the secretion of the different functionality factors of Sertoli cells. It was also distinct after busulfan treatment. The different effect of IL-1β was also pronounced at the level of the functionality marker of Leydig cells (3βHSD). This demonstrated that the effect could be both direct on spermatogonial cells during the different stages and indirect through regulation of Sertoli and Leydig cells producing factors involved in the development of the different stages of spermatogenesis. Previous studies in our laboratory and by others have shown an effect of IL-1β on various activities of Sertoli cells (secretion of transferrin, IL-1 expression, and others) [9] and the division of germ cells [13]. Furthermore, the lower effect of IL-1β on the development of spermatogenesis in busulfan-treated mice compared to normal mice in the MCS may suggest impairment in spermatogonial and supporting cell functionality following chemotherapy treatment.

Our findings indicate a change in IL-1β levels following busulfan treatment compared with the control group, which may suggest the possible involvement of IL-1β in the mechanism of busulfan’s effect on the impairment of spermatogenesis. It also clearly indicates the possibility of IL-1β involvement in testicular physiological activity and pathological conditions. These results may assist in deepening our understanding of the biomolecular mechanisms involved in the control of spermatogenesis and testicular function in physiological and pathological conditions that can cause male fertility impairment and help towards developing future therapeutic strategies in these conditions.

## 4. Material and Methods

### 4.1. Mice

We used 7-day-old sexually immature ICR male mice. The mice were ordered from Envigo Laboratories (Jerusalem, Israel) and were kept by the Guiding Principles for the Care and Use of Research Animals promulgated by the Society for the Study of Reproduction in the animal house of Ben Gurion University (IL-21-03-2020).

### 4.2. Busulfan Treatment

Immature (7-day-old) mice were injected intraperitoneally (IP) with busulfan (BU) (Sigma, St. Louis, MO, USA) (45 mg/kg, 100 µL/mouse) diluted with DMSO and distilled water at a ratio of 1:2. Control mice (CT) were injected with DMSO and distilled water. The mice were sacrificed 1–4, 6, and 10 weeks post injection. Testicular tissues were fixed in Bouin’s solution (Kaltek, Padova, Italy) for histological evaluation, frozen for RNA extraction, or immediately used for cell culture (10 days post BU injection).

### 4.3. Tissue Fixation in Paraffin and Sectioning

After extraction, testicular tissues were placed in Bouin’s solution. Tissues were then treated with ethanol and xylene (BIO-LAB, Hayetzira Jerusalem, Israel) at a temperature of 40 °C, as detailed by Abu Elhija et al. (2012) [28]. Tissues were then embedded in paraffin and stored at 4 °C until use.

To cut the tissues, a microtome was used (Leica Geosystems, Heerbrugg, Switzerland). The cutting thickness was set at 5 μm at an angle of 40°. These layers were first transferred to a 30% ethanol solution before then being transferred to a 37 °C water bath and placed onto slides. The slides were then dried and transferred to an oven (60 °C) overnight.

### 4.4. Hematoxylin and Eosin Staining

The process of staining was performed according to Abumadighem et al. (2018) [2]. The cut of the tissue on the slides was deparaffinized in K-clear solution (Kaltek), rehydrated in ethanol (100%, 70%, and 50%), and then rehydrated with deionized water. Next, the slides were incubated in a hematoxylin reagent (sigma) (1:5 phosphate-buffered saline–PBS) and then rinsed with tap water. This was followed by a short dip in eosin (Kaltek, Padova, Italy) before then being rinsed with distilled water. Slides were then dehydrated with ascending ethanol concentrations of 50%, 70%, and 100%, followed by the use of K-clear. Finally, the slides were dried and covered by a coverslip using Permount (Thermo Fisher Scientific, Denver, CO, USA)

### 4.5. Testicular Tissue Immunofluorescence Staining

After deparaffinization and deionization (as mentioned in H&E staining), antigen retrieval was performed by heating the slides with urea solution (0.36 g/mL) as detailed by Abofoul-Azab et al. (2018) [26]. To block all the non-specific sites, a blocking solution (5% donkey serum Jackson ImmunoResearch Laboratories, West Grove, PA, USA) was used. A primary antibody was diluted with 5% donkey serum, added to the tissues, and incubated overnight. Slides were washed the next day. Thereafter, a secondary antibody diluted with 5% donkey serum was added to tissues. DAPI (4′-6-Diamidino-2-phenylindole, Santa Cruz, CA, USA) was then used to stain the DNA. As a primary antibody for IL-1β, we used polyclonal rabbit antibody (1:250, NB-600-633, NOVUS biologicals, Littleton, CO, USA).

Cy3 (Rabbit 1:700, 711-006-152, 711-006-152, Jackson ImmunoResearch, West Grove, PA, USA) was used as a secondary antibody.

### 4.6. Isolating Cells from Seminiferous Tubules of Testicular Tissues

Testicular tissues were isolated from 7-day-old ICR mice or 10 days post busulfan injection. The seminiferous tubules were isolated from tunics that cover the testis while it is in PBS. Seminiferous tubules were then snipped to extract the cells. The cell extraction was obtained as described by Elhija et al. (2012). A centrifuge (Megafuge 1.0, Heraeus, Hanau, Germany) was then used. Thereafter, the upper fluid containing the cells of the interstitial tissue was removed. Cells were enzymatically extracted by adding collagenase type V and DNase (Sigma, St. Louis, MO, USA) to the precipitate (which contained the seminiferous tubules), with cells then incubated in a 37 °C shaking bathtub.

Isolated cells were filtered through a cell strainer (70 µm; BD Biosciences, San Jose, CA, USA) and centrifuged, with the supernatant then removed and cells suspended with 1 mL of StemPro-34 media (Invitrogen, Thermo Fisher Scientific, Waltham, MA, USA).

### 4.7. Preparation of Slides for Cell Staining

Cells (100,000 cells/100 μL PBS per slide) were fixed with cold methanol (BIO-LAB, Jerusalem, Israel) at (−20 °C).

### 4.8. Immunofluorescence Staining for Cells

The fixed cells were examined for the presence of different spermatogenic cells as described by Sawaied et al. (2021) [29]. For evaluating the premeiotic cells (Vasa), meiotic cells (Boule), and post-meiotic cells (acrosin), immunostaining was performed. First, the slides were dipped in PBS solution for 2 min, followed by blocking and continuation of the steps detailed previously in immunofluorescence staining for tissues (addition of the primary antibody, washing, drying, the addition of DAPI, and covering of the glass with varnish). Primary antibodies: polyclonal Vasa rabbit anti-mouse (1:200, NBP2-24558; Novus Biologicals), mouse monoclonal Boule (1:100, sc-166660, Santa Cruz Biotechnology, Santa Cruz, CA, USA), polyclonal acrosin rabbit anti-mouse (1:100, NBP-14260, Novus Biologicals). Secondary antibodies: Cy3 (mouse 1:500, 715-006-150; rabbit 1:700, 711-006-152, 711-006-152; Jackson ImmunoResearch, West Grove, PA, USA).

### 4.9. D Culture Preparation

A total of 200,000 cells were grown in 500 μL of growth medium per well in a 24-well plate. This growth medium includes 48% StemPro media with Pen-Strep (1%) as an antibiotic, as well as the following growth factors: human rEGF (recombinant epidermal growth factor) (20 ng mL^−1^) (Biolegend, San Diego, CA, USA), human rGDNF (glial cell line-derived nerve growth factor) (10 ng mL^−1^) (Biolegend), mouse rLIF (leukemia inhibitory factor) (10 ng mL^−1^) (Biolegend), and mouse r-bFGF (basic fibroblast growth factor) (10 ng mL^−1^) (Biolegend). The growth medium also included 10% KSR (Gibco, Grand Island, NY, USA) and 42% methylcellulose (R&D, Minneapolis, MI, USA). Recombinant IL-1β (Biolegend) in different concentrations (10, 100, 1000 pg/mL) was added according to the treatment protocol.

### 4.10. Quantitative Real-Time PCR

RNA extraction and complementary DNA synthesis were performed according to the protocol described by AbuMadighem et al. (2018) [2].

qPCR is a method performed using SYBR Green blue dye (PCR Biosystems Ltd., Aztec House, London, UK), the LightCycler 96 real-time PCR machine (Roche, Roche Diagnostics Corporation, Roche CustomBiotech, Indianapolis, IN, USA), and specific primers for each cell marker: GAPDH—Fw-5′-ACCACAGTCCATGCCATCAC Rv-5′-CACCACCCTGTTGCTGTAGCC; Vasa—Fw-5′-AGTATTCATGGTGATCGGGAGC AG Rv-5′-GCAACAAGAACTGGGCACTTTCCA; Boule—Fw-5′-AACCCAACAAGTGGCCCAAGATAC Rv-5′-CTTTGGACACTCCAGCTCTGTCAT; acrosin—Fw-5′-TGTCCGTGGTTGCCAAGGATAACA Rv-5′-AATCCGGGTACCTGCTTGTGAGTT; AR—Fw-5′-TTGGGTGTGGAAGCATTGGA Rv-5′-TGGCGTAACCTCCCTTGAAA; ABP—Fw-5′-GCAGCATGAGGATTGCACTA Rv-5′-CATGAGGCTGGGGAATGTCT; FSHR—Fw-5′-GTGCATTCAACGGAACCCAG Rv-5′-AGGGAGCTTTTTCAAGC GGT; transferrin—Fw-5′-GCAGCATGAGGATTGCACTA Rv-5′-CATGAGGCTGGGGAATGTCT; GDNF—FW-5′-GCCCCTGCTTTCTATCTGCT RW-5′-AGCCTTCTGAATGCGTGGTT; 3β-HSD—Fw-5′-AAGGTGACAGTGTTGGAAGGA Rw-5′-ACAGGCCTCCAATAAGTTCTGG; IL-1β—Fw-5′-CAGGATGAGGACATGAGCACC Rw-5′-CTCTGCAGACTCAAACTCCAG. The reaction consists of 10 μL SYBR Green, 1 μL primer mix (fw + rev), 4 μL cDNA, and 5 μL UPW per well. The relative quantity of the gene expression was analyzed using the 2^−ΔΔCt^ method, and the results were expressed as a fold increase in relation to the GAPDH of the same examined sample.

### 4.11. Data Analysis and Statistical Evaluation

Each culture condition was tested in 4–8 wells. The plotted data are means calculated from 3–10 repeats of the experiment. Statistical significance for qPCR results was tested with a two-tailed, unpaired *t*-test. The standard deviation represents the variability between the independent experiments.

## Figures and Tables

**Table 1:** Summary of the effect of IL-1β on the percentages of spermatogenic cells developed in vitro from cultures of cells isolated from seminiferous tubules of normal and busulfan-treated immature mice.

		VASA	BOULE	ACROSIN
BC	Normal	6.00 ± 1.00	0.00 ± 0.00	0.00 ± 0.00
	BU	2.33 ± 0.33 **	0.00 ± 0.00	0.00 ± 0.00
CT	Normal	12.50 ± 1.26	11.25 ± 0.48	4.00 ± 0.41
	BU	11.00 ± 0.55	8.80 ± 0.73 *	2.60 ± 0.24 *
IL-1β	Normal	22.33 ± 0.66 $$	18.67 ± 1.76 $$	10.67 ± 1.20 $
	BU	20.00 ± 0.63 @@@	14.40 ± 0.75 * @@@	7.60 ± 0.51 * @@@

Mice treatments and in vitro cultures as mentioned in Figure 3, Figure 4 and Figure 5. We summarized and compared results from Figure 4 and 5 related to staining for Vasa, Boule, and acrosin before culture (BC) and after 5 weeks of culture for normal immature mice and busulfan-treated immature mice in the absence (CT) or presence of IL-1β (IL-1β). * indicates the significance of BU-treated mice compared to normal immature mice. $ indicates significance compared to the CT of normal mice. @ indicates significance compared to the CT of BU-treated mice. * *p* < 0.05, ** *p* < 0.01, $ *p* < 0.05, $$ *p* < 0.01, @@@ *p* < 0.001. The green color indicates a significant increase. The red color indicates a significant decrease.

## Data Availability

The data that support the findings of this study are available from the corresponding author upon reasonable request.

## References

[1] Lash B.W., Gilman P.B. (2013). Principles of Cytotoxic Chemotherapy. Cancer Immunotherapy.

[2] AbuMadighem A., Solomon R., Stepanovsky A., Kapelushnik J., Shi Q., Meese E., Lunenfeld E., Huleihel M. (2018). Development of Spermatogenesis In Vitro in Three-Dimensional Culture from Spermatogonial Cells of Busulfan-Treated Immature Mice. Int. J. Mol. Sci..

[3] Meistrich M.L., Finch M., Da Cunha M.F., Hacker U., Au W.W. (1982). Damaging effects of fourteen chemotherapeutic drugs on mouse testis cells. Cancer Res..

[4] JO’Shaughnessy P., Hu L., Baker P.J. (2008). Effect of germ cell depletion on levels of specific mRNA transcripts in mouse Sertoli cells and Leydig cells. Reproduction.

[5] Mahmoud H. (2012). Concise Review: Spermatogenesis in an Artificial Three-Dimensional System. Stem Cells.

[6] Heindel J.J., Treinen K.A. (1989). Physiology of the Male Reproductive System: Endocrine, Paracrine and Autocrine Regulation. Toxicol. Pathol..

[7] Söder O., Sultana T., Jonsson C., Wahlgren A., Petersen C., Holst M. (2000). The interleukin-1 system in the testis. Andrologia.

[8] Huleihel M., Lunenfeld E. (2004). Regulation of spermatogenesis by paracrine/autocrine testicular factors. Asian J. Androl..

[9] Huleihel M., Lunenfeld E. (2002). Involvement of intratesticular IL-1 system in the regulation of Sertoli cell functions. Mol. Cell. Endocrinol..

[10] Petersen C., Fröysa B., Söder O. (2004). Endotoxin and proinflammatory cytokines modulate Sertoli cell proliferation in vitro. J. Reprod. Immunol..

[11] Syriou V., Papanikolaou D., Kozyraki A., Goulis D.G. (2018). Cytokines and male infertility. Eur. Cytokine Netw..

[12] Calkins J.H., Guo H., Sigel M.M., Lin T. (1990). Differential effects of recombinant interleukin-1α and β on leydig cell function. Biochem. Biophys. Res. Commun..

[13] Lysiak J.J. (2004). The role of tumor necrosis factor-alpha and interleukin-1 in the mammalian testis and their involvement in testicular torsion and autoimmune orchitis. Reprod. Biol. Endocrinol..

[14] Qin Y., Liu L., He Y., Wang C., Liang M., Chen X., Hao H., Qin T., Zhao X., Wang D. (2016). Testicular Busulfan Injection in Mice to Prepare Recipients for Spermatogonial Stem Cell Transplantation Is Safe and Non-Toxic. PLoS ONE.

[15] Zhao L., Zhao J., Dong Z., Xu S., Wang D. (2023). Mechanisms underlying impaired spermatogenic function in orchitis induced by busulfan. Reprod. Toxicol..

[16] Ramaswamy S., Weinbauer G.F. (2014). Endocrine control of spermatogenesis: Role of FSH and LH/testosterone. Spermatogenesis.

[17] De Kretser D.M., Loveland K.L., Meinhardt A., Simorangkir D., Wreford N. (1998). Spermatogenesis. Hum. Reprod..

[18] Smith L.B., Walker W.H. (2014). The regulation of spermatogenesis by androgens. Semin. Cell Dev. Biol..

[19] Hagenäs L., Ritzén E.M., Plöen L., Hansson V., French F.S., Nayfeh S.N. (1975). Sertoli cell origin of testicular androgen-binding protein (ABP). Mol. Cell. Endocrinol..

[20] Mruk D.D., Cheng C.Y. (2004). Sertoli-Sertoli and Sertoli-Germ Cell Interactions and Their Significance in Germ Cell Movement in the Seminiferous Epithelium during Spermatogenesis. Endocr. Rev..

[21] Boockfor F.R., Schwarz L.K. (1991). Effects of Interleukin-6, Interleukin-2, and Tumor Necrosis Factor α on Transferrin Release from Sertoli Cells in Culture. Endocrinology.

[22] Gassei K., Orwig K.E. (2016). Experimental methods to preserve male fertility and treat male factor infertility. Fertil. Steril..

[23] Dissanayake D. (2018). In Vitro Spermatogenesis; Past, Present, and Future. Spermatozoa—Facts and Perspectives.

[24] Huleihel M., AbuElhija M., Lunenfeld E. (2007). In vitro culture of testicular germ cells: Regulatory factors and limitations. Growth Factors.

[25] Hunter D., Anand-Ivell R., Danner S., Ivell R. (2012). Models of in vitro spermatogenesis. Spermatogenesis.

[26] Abofoul-Azab M., AbuMadighem A., Lunenfeld E., Kapelushnik J., Shi Q., Pinkas H., Huleihel M. (2018). Development of Postmeiotic Cells In Vitro from Spermatogonial Cells of Prepubertal Cancer Patients. Stem Cells Dev..

[27] Chen X., Liang M., Wang D. (2018). Progress on the study of the mechanism of busulfan cytotoxicity. Cytotechnology.

[28] Abu Elhija M., Lunenfeld E., Schlatt S., Huleihel M. (2012). Differentiation of murine male germ cells to spermatozoa in a soft agar culture system. Asian J. Androl..

[29] Sawaied A., Arazi E., AbuElhija A., Lunenfeld E., Huleihel M. (2021). The Presence of Colony-Stimulating Factor-1 and Its Receptor in Different Cells of the Testis; It Involved in the Development of Spermatogenesis In Vitro. Int. J. Mol. Sci..

